# Longitudinal Outcomes of Individual Placement and Support for Patients With Severe Mental Illness in Italy

**DOI:** 10.3389/ijph.2026.1608701

**Published:** 2026-02-10

**Authors:** Greta Mazzetti, Giulia Paganin, Antonella Mastrocola, Vincenzo Trono, Dina Guglielmi, Angelo Fioritti

**Affiliations:** 1 Department of Education Studies, University of Bologna, Bologna, Italy; 2 Department of Mental Health and Substance Abuse, AUSL Romagna, Ravenna, Italy

**Keywords:** individual placement and support, IPS, mental disorder, occupational health, vocational rehabilitation

## Abstract

**Objectives:**

To assess the long-term impact of the Individual Placement and Support (IPS) model on employment outcomes among individuals with severe mental illnesses (SMIs) and personality disorders (PDs) in Italy, and to examine the role of sociodemographic and clinical factors over a 42-month period.

**Methods:**

We analyzed a 42-month longitudinal cohort of 1,408 IPS participants from seven Community Mental Health Centers in northern Italy. Data on demographics, diagnoses, and employment history were collected. Employment outcomes were compared across diagnostic groups and by nativity using Chi-square and Kruskal-Wallis tests.

**Results:**

Employment rates varied significantly by diagnosis. Participants with schizophrenia, PDs, and depression achieved higher job acquisition rates, whereas those with addiction disorders and milder psychiatric conditions faced greater barriers. Native participants were more likely to obtain employment, although job retention rates were comparable between groups. Job tenure differed across diagnoses, with those with addiction disorders showing shorter employment durations.

**Conclusion:**

The IPS model improves employment for individuals with SMIs and PDs, though disparities by diagnosis and nativity remain. Tailored interventions are needed to promote more inclusive and equitable vocational rehabilitation.

## Introduction

The Individual Placement and Support (IPS) model of supported employment has been increasingly recognized as a key intervention to improve the employment outcomes of individuals with severe mental illness (SMI) and other complex mental health needs. IPS operates on principles that emphasize individualized support, rapid placement into competitive employment, and the integration of employment services with mental health treatment. This evidence-based approach adheres to a “place-then-train” methodology, which prioritizes immediate entry into the workforce over prolonged preparatory training programs. Unlike traditional vocational rehabilitation models, IPS incorporates ongoing support tailored to the specific needs of individuals, helping them navigate both the workplace and their mental health challenges [1, 2].

Data for the present study are drawn from the regional program of Emilia-Romagna, active since 2017, and encompassing all patients receiving IPS services at mental health centers or specialized services for drug addictions. Emilia-Romagna represents a pivotal region in the implementation of IPS in Italy, where mental health and addiction services operate within a robust framework of community care established after the Italian Mental Health Reform of 1978 [3]. This reform facilitated the transition from institutional to community-based mental health services, creating a context conducive to implementing innovative interventions like IPS [4].

The introduction of IPS to Italy dates back to 2003, when Rimini participated as one of six European sites in the EQOLISE trial [5]. The trial demonstrated IPS’s superior effectiveness compared to traditional vocational rehabilitation, with significantly higher rates of employment, income, hours worked, and job retention among participants. Encouraged by these results, the local health trust of Rimini ensured the program’s continuity, maintaining its activities for over two decades. This pilot initiative catalyzed a regional effort in Emilia-Romagna, where IPS has been systematically implemented across all 41 mental health centers since 2010. Approximately 1,000 patients annually participate in IPS programs in the region, supported by embedded specialists who work within multidisciplinary teams.

Emilia-Romagna’s IPS program has become a model for other Italian regions, such as Veneto and Lombardy, further expanding IPS’s reach and impact nationally. The region was the first in Italy to join the International IPS Learning Collaborative in 2012, fostering ongoing quality assurance and professional development through global partnerships. Italy also co-founded the European IPS Learning Collaborative, which continues to meet regularly in Rimini, reflecting the country’s leadership in adapting IPS to diverse populations and contexts [6]. In Emilia-Romagna, the flexibility of IPS has facilitated its expansion into specialized services, including drug addiction centers and adolescent mental health programs in Bologna and Ravenna. Pilot projects in Piacenza for individuals with developmental disorders, and in Bologna for the general unemployed population, highlight the adaptability of IPS in addressing varying needs. The ongoing fidelity monitoring and outcome tracking by the region ensure that implementation aligns with international standards while allowing for local adaptations.

The adaptation of IPS to the Italian context, particularly for individuals with personality disorders (PDs), has highlighted the importance of addressing unique challenges that may influence employment outcomes. Theoretical underpinnings of IPS suggest that its principles, such as personalized support and workplace integration, can be particularly beneficial for individuals with PDs if supplemented with interventions targeting interpersonal skills and emotional regulation [7, 8]. The SCION trial demonstrated that IPS was as effective for individuals with PDs as for those with other psychiatric diagnoses, although modifications such as enhanced skills training could further optimize outcomes.

European policy initiatives, such as the High-Level Conference on Mental Health and Work held in Brussels in 2024, have underscored the role of supported employment models like IPS in promoting social inclusion and economic participation [6]. These initiatives align with broader European Union goals to reduce unemployment among individuals with psychosocial disabilities by creating inclusive labor markets. Such policies advocate for the integration of IPS into comprehensive employment and mental health strategies, ensuring that individuals with mental health challenges receive the support needed to thrive in competitive work environments.

The theoretical and empirical foundations of IPS underscore its potential to transform employment outcomes for individuals with mental health conditions. Studies have consistently demonstrated the model’s effectiveness in diverse populations, provided that implementation fidelity is maintained and adaptations are made to address specific barriers [9]. The integration of cognitive-behavioral techniques and personalized coaching can further support individuals in managing workplace challenges, while the embedding of employment specialists within mental health teams ensures holistic care. Additionally, continuous engagement and tailored interventions have been shown to enhance participant outcomes, emphasizing the need for ongoing innovation in IPS practices [10].

The current study aims to assess the feasibility and outcomes of IPS in individuals with SMIs and PDs in Emilia-Romagna. It focuses on competitive employment outcomes over a 42-month follow-up period and examines the associations between employment success and sociodemographic, clinical, and work history variables. By situating these findings within the broader context of IPS research and European policy frameworks, this study contributes to the growing body of evidence supporting the integration of IPS into mental health systems worldwide. The results are expected to provide valuable insights into optimizing IPS for diverse populations and furthering its impact on social and economic inclusion. Accordingly, the study addresses the following research questions (*RQs*):

*RQ1*: What are the sociodemographic, clinical, and work-history characteristics of individuals enrolled in IPS services across the Emilia-Romagna region?
*RQ2*: What are the employment outcomes achieved during the 42-month follow-up period?
*RQ3*: How are these outcomes associated with participants’ sociodemographic and clinical characteristics, previous work experience, and migrant background?

## Methods

### Data Source and Study Design

The data analyzed in this study were drawn from the regional Individual Placement and Support (IPS) database coordinated by the Emilia-Romagna Department of Mental Health and Pathological Addictions. The database compiles anonymized information on all individuals enrolled in IPS programs across the region’s provincial mental health services, including both community mental health centers and addiction services (*Servizi per le Dipendenze*). Data are entered by trained IPS specialists under the supervision of local coordinators and consolidated at the regional level. The dataset provides longitudinal information on participants’ socio-demographic, clinical, and employment characteristics, as well as details on vocational trajectories over a 42-month observation period. The regional IPS database covers a 42-month observation window but does not include a uniform follow-up length for all participants. Each individual contributed data for the period of their actual IPS participation, from enrolment to the latest available update. Employment information was entered by IPS specialists based on verified employer feedback and routinely validated by local coordinators before regional consolidation.

### Participants

Data for the present study were extracted from the database of the regional program of Emilia-Romagna, active since 2017 and comprising all patients receiving IPS at mental health centers or at specialized services for drug addictions, The sample included 1,408 participants aged between 18 and 64 years (M = 37.77, *SD* = 11.19) at program entry. The gender distribution was nearly balanced, with 53.1% male and 46.9% female participants. The clinical and sociodemographic characteristics of the participants, including age, gender, diagnostic categories, and prior employment experience, are comprehensively detailed in Table 1, which provides an essential baseline for evaluating the effectiveness of the IPS program.

**TABLE 1:** Clinical and Sociodemographic Characteristics of Study Participants (Emilia-Romagna, Italy, 2017–2021).

Characteristic	Percentage (%)	Frequency (n)
Gender		
Male	53.1	748
Female	46.9	660
Age		
Mean (years)	-	37.77 (SD = 11.19)
Primary diagnosis		
Schizophrenia and other psychoses	26.4	372
Personality and behavioral disorders	22.5	317
Depression	17.5	247
Mania and bipolar disorders	12.8	180
Minor disorders (neurotic, stress-related and somatoform disorders)	9.4	133
Addictions	5.5	77
Other psychiatric disorders	4.8	68
Work history		
Previous competitive work experience	22.0	310
Average duration of work experience (years)	-	12.44

All procedures performed in studies involving human participants were in accordance with the ethical standards of the Romagna Ethics Committee (CET-CEROM, Prot. 7506/2023, approved on December 13, 2023) and with the 1964 Helsinki Declaration and its later amendments or comparable ethical standards.

### Procedures and Participant Access to the IPS Service

In the Italian context, IPS services are delivered within the public mental health system as part of a regional initiative coordinated by the Emilia-Romagna Department of Mental Health and Pathological Addictions. Access to the program is based on the principle of “zero exclusion”: all users receiving care from Community Mental Health Centers or addiction services (*Servizi per le Dipendenze*) can be referred to IPS, provided they express an interest in competitive employment. Referrals are typically made by psychiatrists or case managers within multidisciplinary community teams. Participation is entirely voluntary and requires the individual’s informed consent.

After referral, participants meet an employment specialist trained in the IPS model, who collaborates closely with the clinical team. Following IPS principles (Bond et al. [1]; Fioritti et al. [6]), the process starts with a rapid job search and direct placement in the open labor market, rather than through prevocational training (“place-then-train” approach). Employment specialists provide individualized, ongoing support and ensure that clinical and vocational services remain integrated. Contacts with employers are initiated only when explicitly requested by the participant, in order to preserve the principle of competitive employment and respect each individual’s autonomy in the job search process.

The intervention aims to help participants identify their job preferences, obtain and maintain competitive employment, and enhance social inclusion. All IPS services operate under a fidelity monitoring system consistent with the International IPS Fidelity Scale, ensuring adherence to evidence-based standards.

### Inclusion and Exclusion Criteria

All consecutive service users enrolled in IPS through the seven participating Community Mental Health Centers (CMHCs) in Emilia-Romagna were eligible for inclusion, provided they: (i) entered IPS within the continuous 42-month observation window covered by the regional database; (ii) were cared for within adult mental health services; (iii) provided informed consent for the use of anonymized administrative data; and (iv) had, at minimum, baseline sociodemographic and primary diagnosis information recorded. IPS enrolments referred from addiction services integrated within the CMHC network were eligible under the same conditions.

Exclusion criteria were: (i) duplicate records identified during regional data consolidation (removed prior to analysis); (ii) cases outside adult services (e.g., adolescent-only programs not linked to the CMHCs); and (iii) records lacking essential identifiers or the minimum variable set needed for the planned analyses. For outcome analyses, participants with missing employment outcome fields (e.g., job acquisition or job duration) were excluded listwise from the corresponding models; no ad-hoc data imputation was performed. Database lock preceded all analyses.

### Variables and Measures

The following variables from the regional IPS database were included in the analyses:

#### Sociodemographic Variables

- *Gender* (0 = female; 1 = male).
- *Age* (years at IPS entry).
- *Nationality* (0 = Italian; 1 = non-Italian).

#### Clinical Variable

- *Primary diagnosis*, grouped according to the categories reported by treating physicians and used in the regional system: 1) *schizophrenia and other functional psychoses; 2) personality and behavioural disorders; 3) depression; 4) mania and bipolar affective disorders; 5) minor disorders (non-psychiatric and neurotic/somatoform syndromes); 6) addictions; 7) other psychiatric disorders.*

#### Work-History Variable

- *Previous competitive work experience* (0 = none; 1 = at least one period of regular, paid employment in the open labour market prior to IPS enrolment). For descriptive purposes, we report the cumulative duration of previous competitive work (years). This variable refers to participants who had previously held at least one period of regular, paid employment in the open labor market before entering the IPS program, regardless of job type or duration. This information was collected by IPS practitioners during intake and recorded in the regional administrative dataset.

#### Employment Outcomes

- *Job acquisition* (0 = not employed at the time of data collection; 1 = at least one competitive job placement obtained during the 42-month observation period).
- *Job duration* (number of days of employment accrued during the observation period; see Table 2).

**TABLE 2:** Chi-square Analysis of Job Acquisition Frequency Across Diagnostic Categories (Emilia-Romagna, Italy, 2017–2021).

Diagnostic category	Observed (O)	Expected (E)	Standardized residual
Schizophrenia and other functional psychoses	987	552.3	18.49
Mania and bipolar affective disorders	575	552.3	0.97
Depression	734	552.3	7.72
Personality and behavior disorders	871	552.3	13.55
Substance use disorders	184	552.3	−15.66
Minor disorders (non-psychiatric + neurotic/somatoform)	356	552.3	−8.35
Other psychiatric disorders	159	552.3	−16.73
Total	**3,866**	–	–

Observed and expected frequencies refer to the number of job placements assigned to each diagnostic category. Expected values assume an equal distribution across the seven categories. Standardized residuals indicate each category's contribution to the overall chi-square statistic. Bold number (N = 3,866) represents the overall number of observed cases.

### Statistical Analysis

Data were analyzed using the IBM Statistical Package for the Social Sciences (SPSS) for Windows, version 29.0 [11]. Descriptive statistics included mean value ± standard deviation, median, and interquartile range for quantitative variables, while absolute frequencies and percentages were used to represent categorical variables. All tests were two-tailed, with the level of significance set at 0.05. Non-parametric statistics were used due to non-normality in all explorations (Kolmogorov–Smirnov test with Lilliefors significance correction, *p* < 0.05). Between-group comparisons were conducted according to data type (categorical or ordinal) using the Chi-square test, Kruskal-Wallis test for independent samples, and the Mann–Whitney U test.

## Results

All participants underwent clinical assessments conducted by their treating physicians, which identified a wide range of psychiatric diagnoses. Schizophrenia and other functional psychoses were the most common, diagnosed in 26.4% (*n* = 372) of the sample. Personality and behavioral disorders followed at 22.5% (*n* = 317), with depression present in 17.5% (*n* = 247). Additionally, 12.8% (*n* = 180) were diagnosed with mania and bipolar affective disorders, 9.4% (*n* = 133) with minor disorders including non-psychiatric and neuro/somatoform syndromes, 5.5% (*n =* 77) with addictions, and 4.8% (*n* = 68) with other psychiatric disorders. This detailed diagnostic distribution highlights the heterogeneity of the participant population, which is crucial for understanding the varied impacts of the IPS program across different mental health conditions.

A notable portion of the sample, 22%, reported having at least one prior experience with competitive employment, with an average employment duration of 12.44 years (*SD* = 9.73). Results are reported in Table 1. Participants’ previous employment experience may strengthen their engagement with the IPS program and contribute to more favourable employment outcomes. These descriptive findings directly address *RQ1*, showing that the IPS cohort was characterized by substantial clinical heterogeneity and limited prior competitive work experience, which represent the baseline context for interpreting subsequent employment outcomes.

### Between-Group Comparisons

To explore the influence of clinical diagnoses and nationality on employment outcomes, a series of statistical analyses were conducted. The Chi-square test was applied to compare job acquisition rates across different diagnostic categories and between native and non-native participants. The null hypothesis posited that job acquisition would be equally probable across these different categories.

### Job Acquisition: Diagnostic Category Analysis

The analysis revealed significant disparities in job acquisition across the various diagnostic categories (χ^2^ (6) = 1,182.23, *p* < 0.001). Participants diagnosed with schizophrenia, personality disorders, mania and depression secured more jobs than expected under the hypothesis of equal probability. In contrast, those diagnosed with other mental disorders, addictions, and minor disorders secured fewer jobs than anticipated (Table 2). These findings indicate a significant effect of psychiatric diagnosis on employment outcomes within the IPS program, suggesting that certain diagnostic groups may benefit more from IPS than others. Further exploration of these differences reveals that participants with schizophrenia and personality disorders, who often face substantial stigma and barriers to employment, benefitted considerably from IPS, possibly due to the comprehensive support structures offered. The substantial positive deviation from expected job acquisition in these groups highlights IPS’s potential to overcome significant employment barriers. Conversely, the lower-than-expected job acquisition among participants with addictions and minor disorders may indicate a need for specialized support strategies tailored to these groups' unique challenges and treatment needs. These results address *RQ2* by indicating that, although overall job acquisition during the 42-month period was substantial, it varied markedly across diagnostic categories, highlighting unequal employment opportunities within the IPS program.

### Job Acquisition: Nationality Analysis

Nationality was also a significant factor influencing job acquisition (χ^2^(1) = 2,860.90, *p* < 0.001). Native participants were more likely to secure employment compared to non-native participants, highlighting potential disparities in access to job opportunities (Table 3). This finding suggests that non-native participants may encounter additional barriers, such as language difficulties, cultural differences, or discrimination, which can impact their ability to fully benefit from the IPS program. These disparities warrant further investigation into the systemic factors underlying such differences, as well as the development of targeted interventions aimed at promoting equitable access to employment opportunities for all participants. This pattern contributes to answering *RQ3* by showing that migrant background significantly affects employment access, with non-native participants experiencing markedly lower job acquisition rates.

**TABLE 3:** Chi-square Analysis of Job Acquisition Frequency Comparing Native and Non-Native Participants (Emilia-Romagna, Italy, 2017–2021).

Nativity status	Observed (O)	Expected (E)	Standardized residual
Native	3,618	1948.5	37.84
Non-native	279	1948.5	−37.84
Total	**3,897**	–	–

Observed and expected frequencies refer to the number of job placements associated with each nationality category. Expected values are based on an equal distribution across groups. Standardized residuals indicate the contribution of each cell to the chi- square statistic. Bold number (N = 3,897) represents the overall number of observed cases.

### Job Duration: Diagnostic Categories Analysis

The duration of employment was assessed across different diagnostic categories using a Kruskal-Wallis test, which confirmed significant differences in job duration by diagnosis (H(6, *n* = 1,394) = 27.891, *p* < 0.001. As displayed in Table 4, *post hoc* pairwise comparisons (adjusted using Bonferroni correction) showed that participants with addictions had significantly shorter job duration compared with those with bipolar disorders (*p* = 0.027), other psychiatric disorders (*p* = 0.024), depression (*p* = 0.001), and minor disorders (*p* = 0.001).These findings suggest that while individuals with addiction disorders may achieve initial success in maintaining employment, they could face greater difficulties in sustaining long-term job retention, potentially due to relapse risks or ongoing recovery challenges. These findings also inform *RQ3* by demonstrating that job duration is associated with diagnostic category, with participants with addiction disorders showing substantially shorter employment tenures.

**TABLE 4:** Mean Job Duration Across Diagnostic Categories (Emilia-Romagna, Italy, 2017–2021).

Diagnostic category	N	Mean rank
Schizophrenia and other psychoses	372	657.02
Mania and bipolar affective disorders	180	712.74
Depression	247	756.62
Personality and behavioural disorders	317	685.85
Addictions	77	536.36
Minor disorders (non-psychiatric and neurotic/somatoform)	133	772.14
Other psychiatric disorders	68	754.61
Total	**1,394**	-

Higher mean ranks indicate longer job duration. Bold number (N = 1,394) reflects the overall number of cases included in the duration analysis.

### Job Duration: Native Status Analysis

A Mann–Whitney U test was employed to examine differences in job duration between native and non-native participants. The results revealed no significant differences between groups (z = −0.501, *p* = 0.617), suggesting that although disparities exist in job acquisition, once employment is secured, the duration of employment is comparable regardless of nationality (Table 5). This outcome may reflect the effectiveness of IPS support structures in promoting job maintenance across diverse participant backgrounds. These data highlight the complexity of employment retention challenges among diagnostic groups and underscore the need for targeted interventions to enhance long-term job stability where required.

**TABLE 5:** Mann–Whitney Test for Job Duration Comparing Native and Non-Native Participants (Emilia-Romagna, Italy, 2017–2021).

Nativity status	N	Mean rank
Native	1,280	702.78
Non-native	128	721.65
Total	1,408	—

The comparison was conducted using the Mann–Whitney test with native status as grouping variable.

These results further clarify *RQ3* by indicating that, although nativity influences job acquisition, it does not affect job duration once employment is secured.

## Discussion

The present study offers a comprehensive examination of the effectiveness of the Individual Placement and Support (IPS) model in promoting competitive employment among individuals with severe mental illnesses (SMI) and personality disorders (PDs) within the Italian context. By analyzing employment outcomes across different diagnostic categories and nationalities, this research highlights critical factors influencing the success of vocational rehabilitation efforts, providing valuable insights for refining and optimizing IPS interventions.

The study revealed that participants diagnosed with schizophrenia, personality disorders, depression and mania (even thou to a lesser extent), achieved significantly higher job acquisition rates compared to those with other psychiatric conditions. These findings resonate with a substantial body of literature demonstrating that IPS is particularly effective for individuals with severe psychiatric disorders, who often face substantial barriers to employment, including stigma and discrimination [12]. The model’s approach, which prioritizes immediate job placement alongside individualized support, appears to effectively counter these barriers, enhancing employability and facilitating integration into the workforce [13, 14].

The strong employment outcomes among individuals with schizophrenia and personality disorders are particularly noteworthy. These groups are often perceived as among the most challenging to employ due to the chronic and disabling nature of their conditions. However, the findings underscore IPS’s capacity to support these individuals effectively, challenging prevailing assumptions about their employability. The success of IPS in this context may be attributed to its comprehensive support services, which not only address job-specific skills but also provide essential psychosocial support, helping participants manage symptoms and adapt to the workplace environment [15].

Participants with addiction disorders and minor psychiatric conditions exhibited lower job acquisition rates, revealing potential challenges within the IPS framework. For individuals with addiction disorders, factors such as high relapse rates, instability in vocational goals, and the cumulative effects of long-term unemployment and self-stigma may exert greater influence than clinical symptoms alone. Addressing these socio-environmental barriers, alongside integrating specialized addiction counseling and robust relapse prevention strategies, could enhance IPS effectiveness for this group [16]. Similarly, for minor psychiatric conditions such as anxiety or somatoform disorders, prolonged unemployment and social marginalization often outweigh the direct effects of clinical symptoms. These findings suggest a need for IPS to adopt more flexible approaches that address broader social and employment barriers beyond the clinical diagnosis.

Further insights could be gained by examining IPS implementation in alternative settings, such as primary care services in England, where different delivery models might clarify mechanisms underpinning unemployment and inform targeted support strategies [7]. Before drawing definitive conclusions, it is essential to account for potential biases, such as the influence of database duration on outcome measurements. If observed patterns persist, hypotheses regarding low frustration tolerance, frequent relapses, and motivational challenges could inform targeted modifications to IPS interventions for these subgroups.

A significant finding of the present study is the disparity in job acquisition between native and non-native participants, with non-natives securing fewer positions. This result is consistent with previous research indicating that immigrant populations often face heightened barriers to employment, including language difficulties, cultural misunderstandings, and systemic discrimination [17]. These challenges may significantly hinder the effectiveness of standard vocational rehabilitation programs, including IPS. The findings suggest a critical need for IPS adaptations that incorporate culturally sensitive practices and language support services to better meet the needs of non-native participants. Such adaptations could help bridge the gap in employment outcomes and promote equitable access to vocational opportunities [18]. Despite disparities in job acquisition, the study found no significant differences in job retention between native and non-native participants once employment was obtained. This suggests that the comprehensive, personalized support offered by IPS is effective in sustaining employment across diverse cultural and linguistic groups [19]. This result is particularly encouraging, as it implies that while initial barriers to employment access persist, once integrated into the workforce, non-native participants benefit equally from IPS services. These findings reinforce IPS’s potential as an inclusive employment model, provided that entry barriers are adequately addressed [15].

The analysis also identified significant variations in job duration across different diagnostic groups. Participants with addiction disorders experienced notably shorter job durations, emphasizing the complexities involved in sustaining employment during addiction recovery. The risks of relapse and the need for ongoing therapeutic support present significant challenges to continuous employment. These results align with existing literature underscoring the necessity of comprehensive, integrated support systems within IPS to foster job stability for individuals with substance use disorders [20]. Strategies such as enhanced case management, individualized relapse prevention, and closer integration with addiction treatment services may be crucial for extending employment duration among these participants.

In contrast, individuals with depression, schizophrenia and personality disorders demonstrated relatively longer job tenures, suggesting that with appropriate support, sustained employment is achievable even among populations traditionally considered hard to employ. This aligns with previous evidence [21] and challenges the stereotype that individuals with severe psychiatric disorders are inherently unable to maintain stable employment. The success observed likely reflects IPS’s dual emphasis on vocational support and ongoing mental healthcare, enabling participants to manage their conditions effectively while fulfilling work responsibilities [14, 22]. The model’s comprehensive approach appears crucial for fostering both job retention and broader psychosocial stability [23].

The study’s focus on individuals with personality disorders (PDs) provided valuable insights into IPS adaptability. Despite the substantial interpersonal and emotional regulation difficulties associated with PDs, participants achieved commendable employment outcomes. These findings are consistent with broader literature suggesting that tailored vocational interventions, such as IPS, can significantly improve employment prospects for individuals with PDs [2]. The individualized support structure of IPS − emphasizing job matching based on strengths and interests, continuous coaching, and practical problem-solving − appears particularly effective in helping participants manage interpersonal challenges and emotional dysregulation, which are often obstacles to stable employment [24].

The positive outcomes observed emphasize the importance of a comprehensive, person-centered approach in vocational rehabilitation. The integration of employment support with therapeutic interventions aimed at strengthening social skills and emotional resilience is likely a critical factor behind the success of IPS for individuals with PDs. This dual focus supports the development of both vocational competencies and psychosocial functioning, facilitating stable employment and broader community integration [6].

Our findings further support broader European policy initiatives advocating for the integration of mental health and employment services to promote social inclusion and economic participation among individuals with mental health conditions (Council of the European Union, 2023). The consistent success of the IPS model across different European welfare and labor market systems underscores its robustness and adaptability. However, disparities observed across diagnostic groups and between native and non-native participants highlight the importance of continuous evaluation and targeted refinement of IPS practices to ensure that they address the diverse needs of all individuals seeking employment support [25].

### Strengths and Limitations

This study presents several notable strengths. First, it provides a comprehensive analysis of the effectiveness of the Individual Placement and Support (IPS) model within the Italian context, specifically targeting individuals with severe mental illnesses (SMI) and personality disorders (PDs). The large and diverse sample enhances the generalizability of the findings across different diagnostic categories and sociodemographic groups. The longitudinal design, with a follow-up period of 42 months, allows for a detailed examination of employment outcomes over time, providing critical evidence on the durability of IPS-supported employment outcomes. Additionally, the inclusion of a wide range of diagnostic categories, including PDs, contributes to the literature by exploring IPS’s applicability to populations facing unique challenges such as emotional dysregulation and interpersonal difficulties.

Despite these strengths, several limitations should be acknowledged. The reliance on a single geographical region within Italy may limit the generalizability of the findings to areas with different economic and cultural contexts. Furthermore, the observational nature of the study precludes causal inferences; while associations between IPS participation and employment outcomes can be identified, causality cannot be definitively established. Another limitation concerns the potential for selection bias, as individuals who engage with IPS services may differ systematically from those who do not, particularly in motivation, work readiness, and personal skills. Additionally, the absence of a control group limits the ability to compare IPS outcomes directly with those of other vocational rehabilitation models, which could provide a more comprehensive understanding of its relative effectiveness.

### Practical Implications and Future Research

The findings of this study highlight the substantial benefits of Individual Placement and Support (IPS) in enhancing employment outcomes for individuals with severe mental illness (SMI), while emphasizing the need for refinements to address systemic, environmental, and individual barriers. Practical implications include integrating stigma-awareness training for employment specialists and implementing workplace education programs to reduce discrimination and foster inclusivity, both essential for improving job retention and long-term workforce participation [26]. Embedding IPS services into general practice or primary care settings, as demonstrated in some models in England, represents a promising strategy for reaching individuals with milder psychiatric conditions earlier in their treatment trajectory, enabling the simultaneous resolution of complex socio-environmental factors such as long-term unemployment, demotivation, and self-stigma alongside clinical interventions [10].

Tailored strategies, particularly for populations with substance use disorders, could involve the incorporation of relapse prevention measures, motivational interviewing techniques, and specialized addiction support services to address their unique challenges related to job retention and sustained participation [27]. For populations with complex needs, including those with personality disorders, augmented IPS programs integrating cognitive-behavioral therapy, social skills training, or supported education initiatives should be further explored to maximize the potential for stable and meaningful employment outcomes [28]. Building synergies between IPS and social enterprise models may enhance the reach, flexibility, and contextual relevance of supported employment, particularly within community-based mental health systems. These integrated approaches could facilitate broader access to work opportunities while aligning vocational interventions more closely with local social and economic dynamics [29, 30].

Future research should prioritize longitudinal investigations into the sustainability of employment outcomes achieved through IPS, particularly examining how program duration interacts with clinical and non-clinical factors such as stigma, motivation, and self-efficacy [31]. Comparative analyses of IPS implementations across diverse service settings − including primary care, community mental health centers, and specialized addiction services − could offer valuable insights into the adaptability and effectiveness of different delivery models. Additionally, studies focusing on workplace variables, such as supervisor support, job accommodations, and organizational policies, are crucial for understanding how work environments can influence job tenure and facilitate broader recovery for individuals with SMI [32].

Finally, developing standardized, reliable metrics to assess IPS fidelity and its correlation with employment outcomes will be vital for refining best practices, supporting broader implementation, and ensuring that IPS programs remain effective and adaptable across diverse populations and contexts.

### Conclusion

Individual Placement and Support (IPS) continues to offer a robust framework for improving employment outcomes among individuals with severe mental illness. This study contributes long-term observational evidence from the Italian context, demonstrating that IPS can facilitate meaningful workforce participation across a range of diagnostic groups, including those typically facing substantial employment barriers. However, persistent differences related to diagnosis and nationality highlight the need for tailored adaptations within IPS services. Strengthening the responsiveness of vocational rehabilitation models to clinical and social complexity will be essential to ensure more equitable and sustainable employment pathways.

## Data Availability

Data will be available upon reasonable request from the corresponding author.

## References

[1] Bond GR Drake RE Becker DR . An Update on Individual Placement and Support. World Psychiatry (2020) 19(3):390–1. 10.1002/wps.20784 32931093 PMC7491619

[2] Hengartner MP Müller M Rodgers S Rössler W Ajdacic-Gross V . Occupational Functioning and Work Impairment in Association With Personality Disorder Trait-Scores. Soc Psychiatry Psychiatr Epidemiol (2014) 49(3):327–35. 10.1007/s00127-013-0739-2 23835577

[3] Fioritti A . Is Freedom (Still) Therapy? The 40th Anniversary of the Italian Mental Health Care Reform. Epidemiol Psychiatr Sci (2018) 27(4):319–23. 10.1017/S2045796017000671 29335037 PMC6998996

[4] Fioritti A Amaddeo F . Community Mental Health in Italy Today. J Nerv Ment Dis (2014) 202(6):425–7. 10.1097/NMD.0000000000000139 24879570

[5] Burns T Catty J Becker T Drake RE Fioritti A Knapp M The Effectiveness of Supported Employment for People with Severe Mental Illness: A Randomised Controlled Trial. Lancet (2007) 370(9593):1146–52. 10.1016/S0140-6736(07)61516-5 17905167

[6] Fioritti A Jònasson H de Winter L Van Audenhove C van Weeghel J . Mental Health and Work: A European Perspective. Epidemiol Psychiatr Sci (2024) 33:e20. 10.1017/S2045796024000246 38576243 PMC11022246

[7] Juurlink TT Lamers F van Marle HJF Michon H van Busschbach JT Beekman ATF Employment in Personality Disorders and the Effectiveness of Individual Placement and Support: Outcomes From a Secondary Data Analysis. J Occup Rehabil (2020) 30(2):255–62. 10.1007/s10926-019-09868-9 31820219 PMC7293674

[8] Christensen TN Wallstrøm IG Stenager E Bojesen AB Gluud C Nordentoft M Effects of Individual Placement and Support Supplemented With Cognitive Remediation and Work-Focused Social Skills Training for People With Severe Mental Illness: A Randomized Clinical Trial. JAMA Psychiatry (2019) 76(12):1232–40. 10.1001/jamapsychiatry.2019.2291 31483451 PMC6727676

[9] Bond GR Lockett H van Weeghel J . International Growth of Individual Placement and Support. Epidemiol Psychiatr Sci (2020) 29:e183. 10.1017/S2045796020000955 33185176 PMC7681150

[10] Khoronzhevych M Maximova-Mentzoni T Gubrium E Muller AE . Participant Engagement in Supported Employment: A Systematic Scoping Review. J Occup Rehabil (2022) 32(3):414–25. 10.1007/s10926-021-09987-2 34086158 PMC9576634

[11] IBM Corp. IBM SPSS Statistics for Windows, Version 29.0.2.0. Armonk, NY: IBM Corp (2023).

[12] Bond GR Drake RE Pogue JA . Expanding Individual Placement and Support to Populations With Conditions and Disorders Other than Serious Mental Illness. Psychiatr Serv (2019) 70(6):488–98. 10.1176/appi.ps.201800464 30813865

[13] Drake RE Bond GR Mascayano F . Modification of the Individual Placement and Support Model of Supported Employment. Psychiatr Serv (2023) 74(6):656–8. 10.1176/appi.ps.20220484 36718603

[14] Fioritti A Burns T Hilarion P van Weeghel J Cappa C Sunol R Individual Placement and Support in Europe. Psychiatr Rehabil J (2014) 37(2):123–8. 10.1037/prj0000065 24912061

[15] Michon H van Busschbach JT Stant AD van Vugt MD van Weeghel J Kroon H . Effectiveness of Individual Placement and Support for People with Severe Mental Illness in the Netherlands: A 30-Month Randomized Controlled Trial. Psychiatr Rehabil J (2014) 37(2):129–36. 10.1037/prj0000061 24912062

[16] Marsden J Anders P Shaw C Amasiatu C Collate W Eastwood B Superiority and cost-effectiveness of Individual Placement and Support Versus Standard Employment Support for People With Alcohol and Drug Dependence: A Pragmatic, Parallel-Group, Open-Label, Multicentre, Randomised, Controlled, Phase 3 Trial. EClinicalMedicine (2024) 68:102400. 10.1016/j.eclinm.2023.102400 38299044 PMC10828604

[17] Esses VM . Prejudice and Discrimination Toward Immigrants. Annu Rev Psychol (2021) 72:503–31. 10.1146/annurev-psych-080520-102803 32916080

[18] Jónasson H van Weeghel J Koatz D Johnston G Bejerholm U Fioritti A . Boosting the Development of Individual Placement and Support in Europe. Epidemiol Psychiatr Sci (2022) 31:e29. 10.1017/S2045796022000129

[19] Bond GR Drake RE Becker DR . Generalizability of the Individual Placement and Support (IPS) Model of Supported Employment Outside the US. World Psychiatry (2012) 11(1):32–9. 10.1016/j.wpsyc.2012.01.005 22295007 PMC3266767

[20] Harrison J Krieger MJ Johnson HA . Review of Individual Placement and Support Employment Intervention for Persons with Substance Use Disorder. Subst Use Misuse (2019) 55(4):636–43. 10.1080/10826084.2019.1692035 31782349

[21] Drake RE Bond GR Becker DR . Individual Placement and Support: An Evidence-based Approach to Supported Employment. Oxford: Oxford University Press (2012).

[22] Drake RE Bond GR . Individual Placement and Support: History, Current Status, and Future Directions. Psychiatry Clin Neurosci Rep (2023) 2(3):e122. 10.1002/pcn5.122 38867819 PMC11114326

[23] Brinchmann B Widding‐Havneraas T Modini M Rinaldi M Moe CF McDaid D A Meta‐Regression of the Impact of Policy on the Efficacy of Individual Placement and Support. Acta Psychiatr Scand (2020) 141(3):206–20. 10.1111/acps.13129 31733146

[24] Larcombe E Müller A . The Relationship Between Borderline Personality Disorder and Occupational Participation: An Integrative Review. Int J Ment Health Nurs (2022) 31(5):1141–50. 10.1111/inm.13014 35536729

[25] Perkins R Patel R Willett A Chisholm L Rinaldi M . Individual Placement and Support: Cross-Sectional Study of Equality of Access and Outcome for Black, Asian and Minority Ethnic Communities. BJPsych Bull (2022) 46(1):10–5. 10.1192/bjb.2021.9 33583477 PMC8914809

[26] Janssens KME Joosen MCW Henderson C Bakker M den Hollander W van Weeghel J Effectiveness of a Stigma Awareness Intervention on Reemployment of People With Mental Health Issues/Mental Illness: A Cluster Randomised Controlled Trial. J Occup Rehabil (2024) 34(1):87–99. 10.1007/s10926-023-10129-z 37439945 PMC10899371

[27] Hellström L Pedersen P Christensen TN Wallstroem IG Bojesen AB Stenager E Vocational Outcomes of the Individual Placement and Support Model in Subgroups of Diagnoses, Substance Abuse, and Forensic Conditions: A Systematic Review and Analysis of Pooled Original Data. J Occup Rehabil (2021) 31:1–12. 10.1007/s10926-021-09960-z 33661452

[28] Oude Geerdink E Huysmans MA van Kempen H Maarleveld JM van Weeghel J Anema JR . Process Evaluation of Individual Placement and Support and Participatory Workplace Intervention to Increase the Sustainable Work Participation of People with Work Disabilities. J Occup Rehabil (2024) 35:1–11. 10.1007/s10926-024-10214-x 38918334 PMC12089157

[29] Fioritti A D’Alema M Barone R Bruschetta S . Social Enterprises, Vocational Rehabilitation, Supported Employment: Working on Work in Italy. J Nerv Ment Dis (2014) 202(6):498–500. 10.1097/NMD.0000000000000150 24821277

[30] Fioritti A Peloso PF Percudani M . “We Can Work It Out”: The Place of Work in Italian Psychosocial Rehabilitation. Int J Ment Health (2016) 45(1):51–8. 10.1080/00207411.2015.1129841

[31] Adamus C Richter D Sutor K Zürcher SJ Mötteli S . Preference for Competitive Employment in People With Mental Disorders: A Systematic Review and Meta-Analysis of Proportions. J Occup Rehabil (2024) 35:1–16. 10.1007/s10926-024-10192-0 38662329 PMC12089194

[32] Vaingankar JA Teh WL Roystonn K Goh J Zhang YJ Satghare P Roles, Facilitators and Challenges of Employment Support Specialists Assisting Young People With Mental Health Conditions. J Occup Rehabil (2021) 31:405–18. 10.1007/s10926-020-09930-x 33090356 PMC8172398

